# A Co-Precursor Approach Coupled with a Supercritical Modification Method for Constructing Highly Transparent and Superhydrophobic Polymethylsilsesquioxane Aerogels

**DOI:** 10.3390/molecules23040797

**Published:** 2018-03-30

**Authors:** Chaoshuai Lei, Junning Li, Chencheng Sun, Hailong Yang, Tao Xia, Zijun Hu, Yue Zhang

**Affiliations:** 1 School of Materials Science and Engineering, Beihang University, Xueyuan Road 37, Beijing 100191, China; leichaoshuai@126.com (C.L.); xiatao@buaa.edu.cn (T.X.); 2 National Key Laboratory of Advanced Functional Composite Materials, Aerospace Research Institute of Materials and Processing Technology, Beijing 100076, China; ljn1212@163.com (J.L.); sunyahua2005@163.com (C.S.); yhl20032003@126.com (H.Y.)

**Keywords:** polymethylsilsesquioxane aerogels, co-precursor, supercritical modification, transparency, superhydrophobicity

## Abstract

Polymethylsilsesquioxane (PMSQ) aerogels obtained from methyltrimethoxysilane (MTMS) are well-known high-performance porous materials. Highly transparent and hydrophobic PMSQ aerogel would play an important role in transparent vacuum insulation panels. Herein, the co-precursor approach and supercritical modification method were developed to prepare the PMSQ aerogels with high transparency and superhydrophobicity. Firstly, benefiting from the introduction of tetramethoxysilane (TMOS) in the precursor, the pore structure became more uniform and the particle size was decreased. As the TMOS content increased, the light transmittance increased gradually from 54.0% to 81.2%, whereas the contact angle of water droplet decreased from 141° to 99.9°, ascribed to the increase of hydroxyl groups on the skeleton surface. Hence, the supercritical modification method utilizing hexamethyldisilazane was also introduced to enhance the hydrophobic methyl groups on the aerogel’s surface. As a result, the obtained aerogels revealed superhydrophobicity with a contact angle of 155°. Meanwhile, the developed surface modification method did not lead to any significant changes in the pore structure resulting in the superhydrophobic aerogel with a high transparency of 77.2%. The proposed co-precursor approach and supercritical modification method provide a new horizon in the fabrication of highly transparent and superhydrophobic PMSQ aerogels.

## 1. Introduction

Silica aerogels have gained extensive attention in the past decades owing to their extraordinary properties, such as their low density, nanoporous structure, high specific surface area, high optical transparency, and extremely low thermal conductivity in some chemical systems [1]. For these unique characteristics, silica aerogels are presently of interest in thermal insulation [2], acoustic insulation [3], energy-saving windows [4], catalysts [5], and drug delivery systems [6]. Generally, silica gels are synthesized from tetra-alkoxysilane precursors through the sol-gel route and the monolithic aerogels are obtained after draining the pore liquid with supercritical drying [7,8,9]. However, because of the brittle tetra-functional siloxane network, most of the previously reported silica aerogels are usually fragile, hydrophilic, or opaque.

Scientists have made great efforts to improve the properties of silica aerogels. One of the promising approaches was utilizing alkyltrialkoxysilane instead of traditional tetra-functional silicon alkoxides such tetramethylorthosilicate (TMOS) or tetraethylorthosilicate (TEOS) [10,11]. As one kind of alkyltrialkoxysilane, MTMS was usually utilized as the precursor while the obtained flexible and hydrophobic aerogels were opaque due to the phase separation [12,13]. Kanamori et al. reported the first successful fabrication of transparent monolithic PMSQ aerogels by exploiting appropriate surfactants to prevent the macroscopic phase separation [14]. However, the substituent methyl groups in MTMS have steric effects that provide barriers for hydrolysis and condensation reactions of Si–OCH_3_ groups in the sol-gel process [15]. Further hydrolysis and condensation of unhydrolyzed groups during the temperature supercritical drying process will lead to the pore structure coarsening with larger particle and pore size. This will result in the low transparency according to the Rayleigh scattering mode [16,17]. One of the prevalent approaches to improve the transparency of the aerogels was introducing TMOS together with MTMS in a sol-gel route, namely the co-precursor method [18,19]. Benefiting from the low energy barriers, hydrolysis and condensation reactions of TMOS are complete [20,21]. Then the particles in aerogels remain small and uniform after high temperature supercritical drying process, which is beneficial to the enhancement of the transparency of aerogels.

The superhydrophobicity of the aerogels were usually achieved using the surface modification of the skeleton with specific chemical groups such as hexamethyldisilazane (HMDZ). The modification procedure can be performed during the aging process or supercritical drying process or carried out as a post-treatment by gas phase reactions with the formed aerogel network [22,23,24]. The lengthy and tedious aging modification usually took several days or weeks due to the solvent-exchange process [25]. Gas phase modification of the as-prepared aerogels had a negative effect on the pore structure due to the gas condensation. In contrast, supercritical modification technology during the drying process not only rendered modification much faster and more complete but also caused very slightly differences in the pore structure [26,27]. Incorporating hydrophobic groups with the hydroxyl on the silica surface, the supercritical modification would endow the monolithic aerogels with hydrophobicity [23].

In this work, highly transparent PMSQ aerogels were prepared by the co-precursor method. With the increase of TMOS content in the precursor, the particle size and pore diameter of the PMSQ aerogel were decreased gradually due to the relatively complete hydrolysis and condensation reactions. The light transmittance of the obtained aerogels was up to 80%. Meanwhile, superhydrophobicity of the aerogel was realized by modifying the surface hydroxyl groups with hexamethyldisilazane through supercritical drying process. The modified aerogels with a contact angle of 155° retained almost the same pore structure compared with the aerogels without modification. Therefore, the PMSQ aerogel possesses a high transparency of 77.2%. The highly transparent, superhydrophobic aerogels would extend their practical application such as transparent insulation window.

## 2. Materials and Methods

### 2.1. Materials

Methyltrimethoxysilane (MTMS), tetramethoxysilane (TMOS) and hexamethyldisilazane (HMDZ) were obtained from Adamas Reagent Co., Ltd. (Shanghai, China). Acetic acid, ethanol, and ammonia (NH_3_·H_2_O) were purchased from Beijing Chemical Works (Beijing, China). Surfactant n-hexadecyltrimethylammonium chloride (CTAC) was supplied by Sinopharm Chemical Reagent Co., Ltd. (Shanghai, China). All the chemical reagents were analytical reagents and used as received without further purification.

### 2.2. Preparation of Silica Aerogels

Silica hydrogels were synthesized with MTMS and TMOS as co-precursors through a two-step, acid-base catalyzed sol-gel process. In a typical synthesis, 60 mL of diluted aqueous acetic acid (5 mM), 0.6 g of surfactant CTAC were mixed in a glass beaker. MTMS and TMOS were subsequently added with vigorous stirring 30 min for hydrolysis reaction. After that, NH_3_·H_2_O dissolved in 30 mL deionized water was poured into the mixed solution under stirring. The molar ratio of the starting materials was (MTMS + TMOS):H_2_O:acetic acid:CTAC:NH_3_·H_2_O = 1:24:1.4 × 10^−3^:8.9 × 10^−3^:3.8 × 10^−3^. The temperature of hydrolysis and condensation steps was 30 °C. The obtained hydrogels were then aged for 3 days to complete the condensation at 60 °C, followed by washing with ethanol three times (8 h each time) to remove the residual surfactant and chemicals. The washed wet gels were dried by ethanol supercritical method at 255 °C and 8 MPa. The proportion of TMOS (x) in precursor was varied from 0% to 50% in increments of 10%. The obtained aerogels were labeled as T0, T10, T20, T30, T40, and T50, respectively. For the sample T30, the supercritical fluid modification was implemented using HMDZ/ethanol solution with volume ratio of 1:50. The sample was marked as T30-H.

### 2.3. Characterization of Materials

The bulk density of the aerogels was measured from the weight/volume ratio of the monoliths. The microstructure of the aerogels was studied using field-emission scanning electron microscopy (FE-SEM, S-4800, Tokyo, Japan).

Specific surface area and pore size distribution (PSD) were obtained from N_2_ adsorption-desorption (Autosorb-iQ, Boynton Beach, FL, USA). The samples were outgassed at 150 °C prior to the adsorption-desorption measurement. The specific surface areas of the aerogels were calculated by the Brunauer–Emmett–Teller (BET) method, based on the amount of N_2_ adsorbed at pressure 0.05 < P/P_0_ < 0.3. The pore size distributions of the samples were determined by desorption branch of the isotherm by employing the BJH method.

For light transmittance measurements, a UV-vis spectrometer UV-3600 (Shimadzu Corp., Kyoto, Japan) was employed. The obtained transmittance data at 550 nm was normalized into those corresponding to the thickness of 10 mm using the Lambert–Beer equation.

The surface wettability of PMSQ aerogels was evaluated by static contact angle measurement, using a Contact Angle System OCA (Dataphysics, Stuttgart, Germany). A droplet (1 µL) of deionized water was placed very slowly on the surface of the hydrophobic aerogel.

The chemical composition of silica aerogels was characterized with Fourier transform-infrared spectroscopy with the attenuated reflection model (FTIR-ATR, Perkin-Elmer, Waltham, MA, USA).

Compression tests were performed on a monolithic cylindrical sample using a Universal Testing System (CTM5105, Guangzhou, China). The sample was placed between the testing plates and was compressed at a speed of 1 mm/min to 50% strain and this position held for 10 s before the piston was retracted. Three replicates were performed for different samples in one group. The resilience of the aerogels is defined as the ratio of the final dimension after release of compressive stress to the original scale.

## 3. Results and Discussion

### 3.1. Structural Characterization of the PMSQ Aerogels Prepared by the Co-Precursor

**Figure 1:**
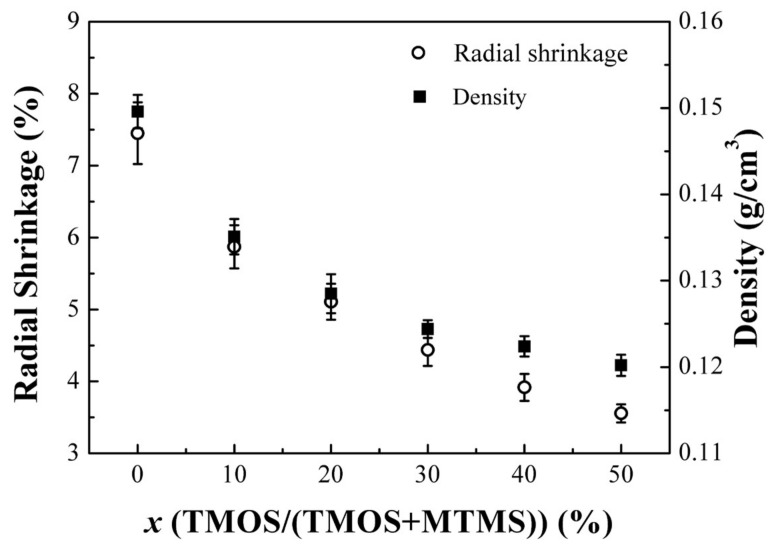
The radial shrinkage and density of aerogels with varied amount of TMOS.

Figure 1 shows the influence of TMOS content on the radial shrinkage and density of the PMSQ aerogels. With the increase of TMOS content in the precursor from 0% to 50%, the radial shrinkage of the aerogels decreases from 7.45% to 3.56%. Correspondingly, the density of the aerogel decreases from 0.150 g/cm^3^ to 0.120 g/cm^3^. Shrinkage of the aerogels during the high temperature supercritical drying process was resulted from the rearrangement reactions in the gel network, which is ascribed to the further hydrolysis and condensation of unhydrolyzed Si–OCH_3_ groups. For the aerogel T0 obtained from MTMS as the sole precursor, the steric effect of the methyl group provided barriers for hydrolysis and condensation reactions [15]. Continued reactions during high temperature drying process caused the PMSQ aerogel to have high radial shrinkage of 7.45%. The reaction of TMOS is more complete in the sol-gel process because of the low energy barriers in hydrolysis and condensation reactions [28]. As a result, the radial shrinkage of the aerogel T50 decreased to 3.56% when the TMOS content was 50% in the precursor.

**Figure 2:**
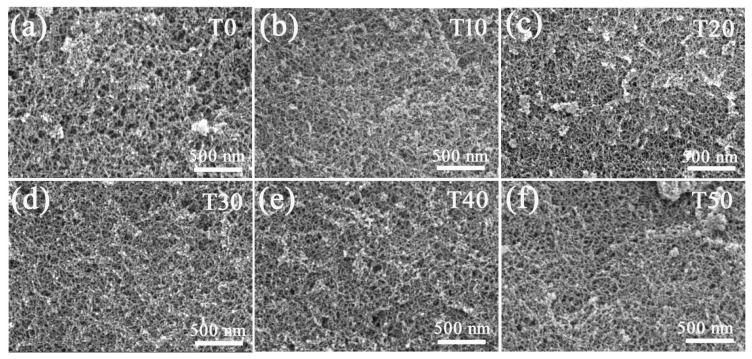
SEM images of the aerogel samples; (**a**) T0, (**b**) T10, (**c**) T20, (**d**) T30, (**e**) T40, and (**f**) T50.

**Figure 3:**
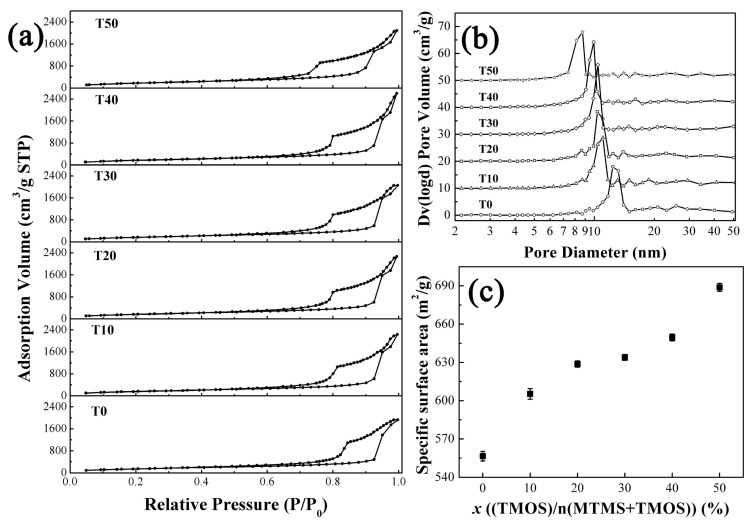
N_2_ adsorption-desorption isotherms (**a**); the pore size distributions (**b**) and the specific surface area of the aerogels (**c**).

Figure 2 presents the SEM images of the aerogels prepared with different contents of TMOS. All the aerogels exhibit 3D continuous network structure which is comprised of nano-scale particles and pores. It is found that the particle size and pore diameter in the aerogels prepared via the co-precursor method are smaller than those in the aerogel T0. Meanwhile, more uniform pore structure was obtained via the co-precursor method. Pore structures were further confirmed in Figure 3. N_2_ adsorption-desorption isotherms in Figure 3a reveal type IV nature and H1 hysteresis loops according to the IUPAC classification, demonstrating that all the aerogels are typical mesoporous materials distributions [29]. The average pore size distributions in Figure 3b reveal that the pore diameter was decreased from 13 nm of T0 to 8.7 nm of T50. A gradual increase in the specific surface areas with increasing TMOS content is depicted in Figure 3c. Aerogel T50 has the highest specific surface area of 688.6 m^2^/g, which is primarily attributed to the smaller particles in the aerogel [30]. While the specific surface area of aerogel T0 is only 556.5 m^2^/g. As we know, less-polar MTMS oligomers are incompatible with any low-molecular weight solvents [14]. Then, surfactants such as CTAC always serve as a structure directing agent to realize mesoporous structure in the aerogels through inhibiting phase separation [31]. The introduced TMOS is soluble in the solvent water due to the rapid hydrolysis reaction [21]. The homogeneous solution in the sol-gel process contributes to the formation of small particles and uniform pore structure. The differences in chemical composition and pore structure will result in differences in the optical transparency, hydrophobicity, and mechanical properties of the aerogels.

### 3.2. The Properties of the PMSQ Aerogels Prepared by the Co-Precursor

#### 3.2.1. The Transparency of Aerogels with Varied TMOS Content

**Figure 4:**
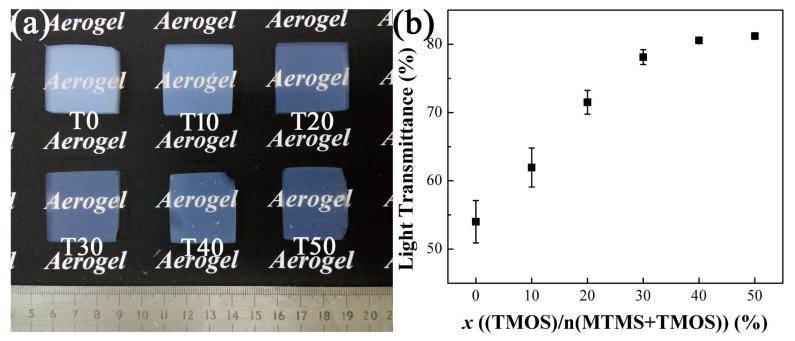
Photographs of the monolithic aerogels (**a**) and the light transmittance at 550 nm wavelength (**b**).

The photographs of the aerogel monoliths are shown in Figure 4a. Obviously, the transparency of the aerogels is improved significantly after the addition of TMOS. Light transmittance in Figure 4b increases from 54.0% of T0 to 81.2% of T50, which is comparable to the value of traditionally derived silica aerogels [32]. In fact, the light transmittance of aerogel T30 is already up to 78.1%, where the molar ratio of TMOS is 30%. The dominant light scattering mode is Rayleigh scattering in the mesoporous aerogels and the particles and pores act as scattering centers [19]. Smaller particle size and pore diameter can decrease the scattering of light, leading to the transmittance increase as predicted by the scattering mode. The co-precursor method with introduction of TMOS plays a significant role in reducing particle size and improving the transparency of PMSQ aerogels.

#### 3.2.2. The Hydrophobicity of Aerogels with Varied TMOS Content

**Figure 5:**
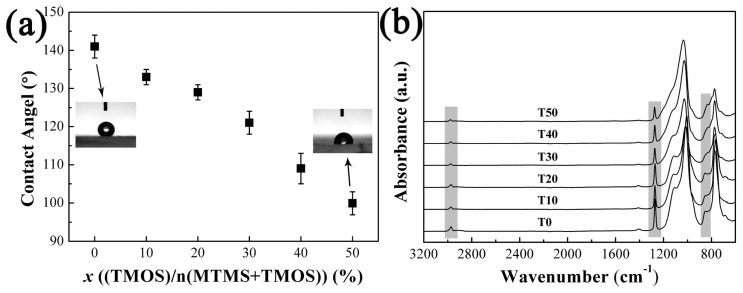
The contact angle of water droplet (**a**) and FTIR-ATR spectra of all the samples (**b**).

The hydrophobicity of aerogels is very importance for their wide used in practical applications [33]. However, the hydrophobicity of the aerogels will decrease with increasing TMOS due to the increase of hydroxyl group on the particle surface. The contact angle of the water droplet in Figure 5a decreased from 141° to 99.9° when the molar ratio of TMOS in precursor increased from 0% to 50%. Chemical structures in the aerogels have been confirmed by FTIR-ATR spectroscopy as shown in Figure 5b. The peaks at 2980 cm^−1^ and 1270 cm^−1^ represent the stretching of the Si–CH_3_ [34]. The stretching vibration peak of Si–C can be observed at 840 cm^−1^ [35]. Notably, the characteristic peaks of Si–CH_3_ gradually become weaker with the increase in TMOS content. Results state that the methyl group in the aerogel skeleton is gradually replaced by the hydroxyl group with the introduction of TMOS, which results in decreased hydrophobicity. Surface modification should be carried out to improve the hydrophobicity of PMSQ aerogels.

#### 3.2.3. The Mechanical Properties of Aerogels with Varied TMOS Content

**Figure 6:**
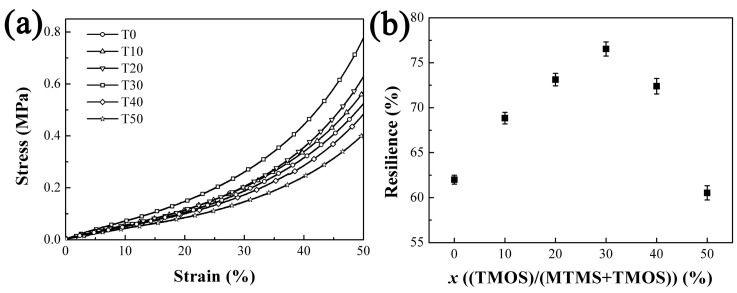
Compressive stress versus strain curves of the PMSQ aerogels (**a**) and the resilience of the samples compressed to 50% strain (**b**).

The mechanical properties of the aerogels were evaluated by uniaxial compression tests. The stress–strain curves in Figure 6a indicate that these PMSQ aerogels could endure ~50% strain without any damage, illustrating that the skeleton remains flexible in the aerogels obtained from the co-precursor method. The compressive strength is enhanced gradually with increasing the molar ratio of TMOS to 30%. Then aerogel T30 possesses the maximum compressive strength of 0.76 MPa at 50% strain. However, further increase in the proportion of TMOS in precursor lead to a significant reduction in the compression strength. The compressive strength of aerogel T50 decreases to 0.4 MPa which is lower than the value of aerogel T0. Meanwhile, the resilience of the aerogels increased with increasing TMOS proportion to reach a maximum value of 76% at sample T30, from which it decreased to 60% of T50, as shown in Figure 6b. The improvement of mechanical properties is derived from the increase of silanol groups that are closely located to each other in the skeleton [14]. The tetra-functional siloxane network would improve the rigidity of the skeleton. However, excess introduction of TMOS can reduce the methyl groups significantly and make the skeleton become brittle. When stress is applied to the aerogel skeleton, the irreversible deformation would damage the framework. Furthermore, the repulsive interactions between hydrophobic moieties will be weakened with the decrease of methyl groups. Therefore, the mechanical properties of aerogel T30 are superior to those of the other aerogels.

### 3.3. The Supercritical Modification of the PMSQ Aerogel

**Figure 7:**
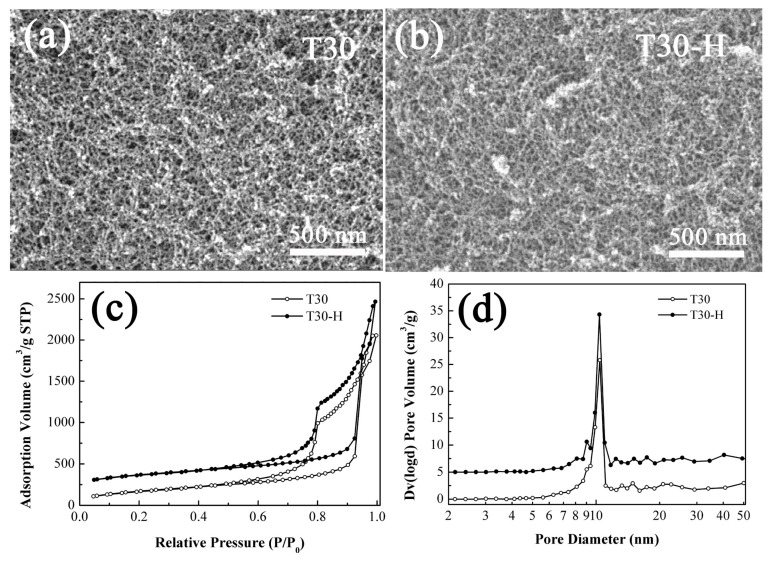
The SEM images of the aerogels T30 and T30-H (**a**,**b**); N_2_ adsorption-desorption isotherms (**c**) and the pore size distributions (**d**) of the aerogels T30 and T30-H.

In order to obtain high-transparency, superhydrophobic, and elastic PMSQ aerogels, the wet gels of T30 were chosen for supercritical fluid modification with hexamethyldisilazane. The modifier HMDZ would react with the –OH on the skeleton surface, which results in methyl coatings on the nanoparticles. Therefore, the condensation of the neighboring –OH on the silica surface during drying is effectively restricted. Then, shrinkage of silica wet gels during drying are effectively inhibited. After saturation with methyl groups, the aerogel surface cannot be covalently attached by a chemical bond [23]. Structural comparison of aerogel T30 and modified aerogel T30-H is shown in Figure 7. It is worth noting that these two samples hold the same uniform pore structure with small particles and pore size, as shown in Figure 7a. The N_2_ adsorption-desorption isotherms and pore size distributions display that pore structures of T30-H are affected very slightly by the methyl groups loading on the aerogel skeleton surface.

**Figure 8:**
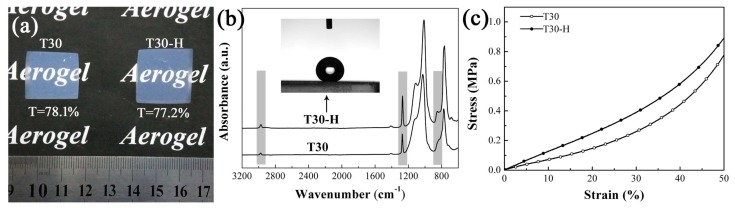
Photographs of the monolithic aerogels T30 and T30-H (**a**); FTIR-ATR spectra (**b**) and compressive stress versus strain curves of aerogels T30 and T30-H (**c**).

There is no significant difference in microstructure between the modified aerogel T30-H and the unmodified aerogel T30. Aerogel T30-H retained a high transparency of 77.2%, which is comparable to the value of aerogel T30 as shown in Figure 8a. The contact angle of T30-H was determined to be 155°, which is indicative of superhydrophobicity. The chemical structure analysis in Figure 8b further supports the covalent bonding of the modifier with the aerogel surface. FTIR-ATR spectroscopy shows that the characteristic peaks of Si–CH_3_ (2980 cm^−1^, 1270 cm^−1^ and 840 cm^−1^) obviously become stronger with the modification process, which signifies the reaction between HMDZ and surface –OH groups. Besides, the –CH_3_ coating endows aerogel T30-H with higher mechanical properties, compared with the aerogel without modification. The compressive strength of aerogel T30-H is 0.90 MPa and the resilience of the aerogel is 85%. This improvement in the mechanical properties is attributed to the repulsive interactions between the surface –CH_3_ groups, which promote the spring-back effect. These characteristics of high transparency, superhydrophobicity, and elasticity are important for the practical application of these materials, such as in transparent vacuum insulation panels.

## 4. Conclusions

Highly transparent, superhydrophobic, and elastic PMSQ aerogels have been prepared via the co-precursor and supercritical modification method. The introduction of TMOS plays an important role in the structural stability of the aerogel during the high temperature supercritical drying process. With the increase in TMOS content, the particle and pore size of the PMSQ aerogel gradually become smaller. The light transmittance increases to 78.1% when the molar ratio of TMOS is 30%. The superhydrophobicity of the aerogel is achieved by reacting the surface hydroxyl groups with HMDS through supercritical fluid modification. Modified aerogels revealed a high contact angle of 155°. The mechanical properties of the aerogels were slightly improved due to the repulsive interactions between the methyl groups. More importantly, the pore structures of PMSQ aerogels with and without modification stay the same. Therefore, the modified aerogel retains a high transparency of 77.2%. These extraordinary characteristics will extend the range of applications for PMSQ aerogels.
